# Endovascular repair for thoracic aortic pseudoaneurysm induced by pedicle screw implantation: a case report with 8 years follow-up

**DOI:** 10.1186/s13019-024-02820-w

**Published:** 2024-06-07

**Authors:** Shufen Zhou, Hui Han, Yidan Zhang, Chang Shu, Mingyao Luo

**Affiliations:** 1 State Key Laboratory of Cardiovascular Disease, Center of Vascular Surgery, Fuwai Hospital, National Center for Cardiovascular Diseases, Chinese Academy of Medical Sciences and Peking Union Medical College, Beijing, 100037 China; 2 Department of Vascular Surgery, Henan Cardiovascular Disease Center, Central-China Branch of National Center for Cardiovascular Diseases, Fuwai Central-China Hospital, Central China Fuwai Hospital of Zhengzhou University, Zhengzhou, 450046 China; 3 Department of Vascular Surgery, Fuwai Yunnan Cardiovascular Hospital, Affiliated Cardiovascular Hospital of Kunming Medical University, Kunming, 650102 China; 4 The Second Hospital of Jilin University, Changchun, 130041 China

**Keywords:** Iatrogenic aortic injury, Pedicle screw fixation, Endovascular repair, Pseudoaneurysm, Penetration

## Abstract

**Background:**

Pedicle screw instrument surgeries can result in the development of aortic pseudoaneurysm, which is a rare yet potentially severe complication; therefore, the purpose of this work is to describe the case of pseudoaneurysm of the thoracic aorta caused by the severe migration of a pedicle screw after surgery.

**Case presentation:**

We herein report a patient who underwent endovascular repair for the pseudoaneurysm of the descending thoracic aorta following thoracic vertebral fixation surgery. A 28–80 mm covered stent was initially inserted through the right femoral artery, and intraoperative aortography revealed a minor extravasation of contrast material. Subsequently, an additional 28–140 mm covered stent was implanted. The patient recovered well during the 8-year follow-up period.

**Conclusions:**

Vascular complications resulting from spinal surgery are severe and rare, necessitating early diagnosis and intervention.

## Background

Iatrogenic aortic pseudoaneurysm caused by pedicle screw instrument surgeries is a rare but potentially serious complication. Injuries to the aorta or iliac arteries can result in a mortality as high as 61% [5]. In the past, lumbar intervertebral disc surgery was a primary cause of iatrogenic injury to the aorta during spinal operation. Literature frequently documented injuries to the proximal iliac vessels. The most common vessel affected was the left common iliac artery, located anteriorly to the L4-L5 intervertebral disc space [5]. Injuries to thoracic aorta is rarely reported during pedicle screw instrumentation surgeries. Herein we describe endovascular solutions for a patient whose displaced screw led to a pseudoaneurysm of the descending thoracic aorta.

## Case presentation

**Fig. 1:**
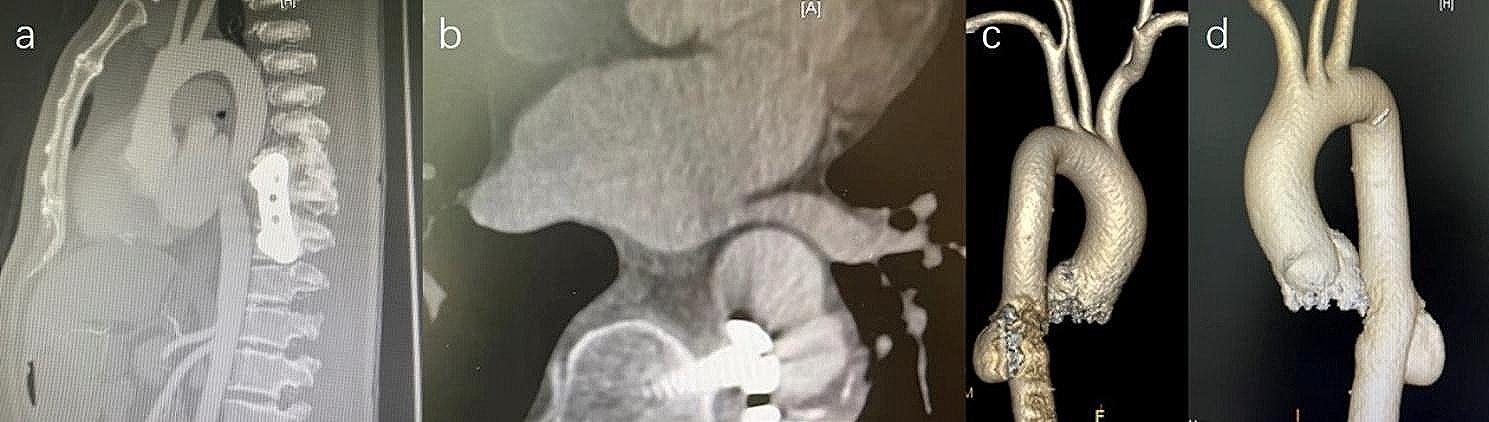
Sagittal (**A**) and axial (**B**) CT angiography showing the malpositioned pedicle screw abutting the descending aorta. CT angiography three-dimensional reconstruction (**C** and **D**) confirmed the formation of a pseudoaneurysm

A 51-year-old man had previously undergone a vertebral body resection, bone grafting and internal fixation surgery due to eosinophilic granuloma of the 9th thoracic vertebra in Dec 2014. In June 2015, the patient presented to our hospital with symptoms of chest tightness and lower back discomfort. Computed tomography (CT) scan (Fig. 1) revealed a pseudoaneurysm of the thoracic aorta and recommended surgical intervention. However, the patient declined the surgery because he considered the risk, especially paraplegia, to be high. Six months later, follow-up CT examination revealed no improvement in the condition of the pseudoaneurysm, leading to the decision to seek further treatment at our hospital in Jan 2016.

Preoperative X-rays showed an abnormal bulging shadow in the descending aorta and a slightly enlarged aortic arch. CT scans revealed that a screw used in spinal surgery penetrated the wall of the descending aorta and locally formed a pseudoaneurysm, with the size of 27*42 mm, in the mid-segment of the descending aorta, with a significant amount of intraluminal thrombus and shell-like calcifications. The proximal landing diameter is approximately 23 mm, and the distal landing diameter is approximately 22 mm.

**Fig. 2:**
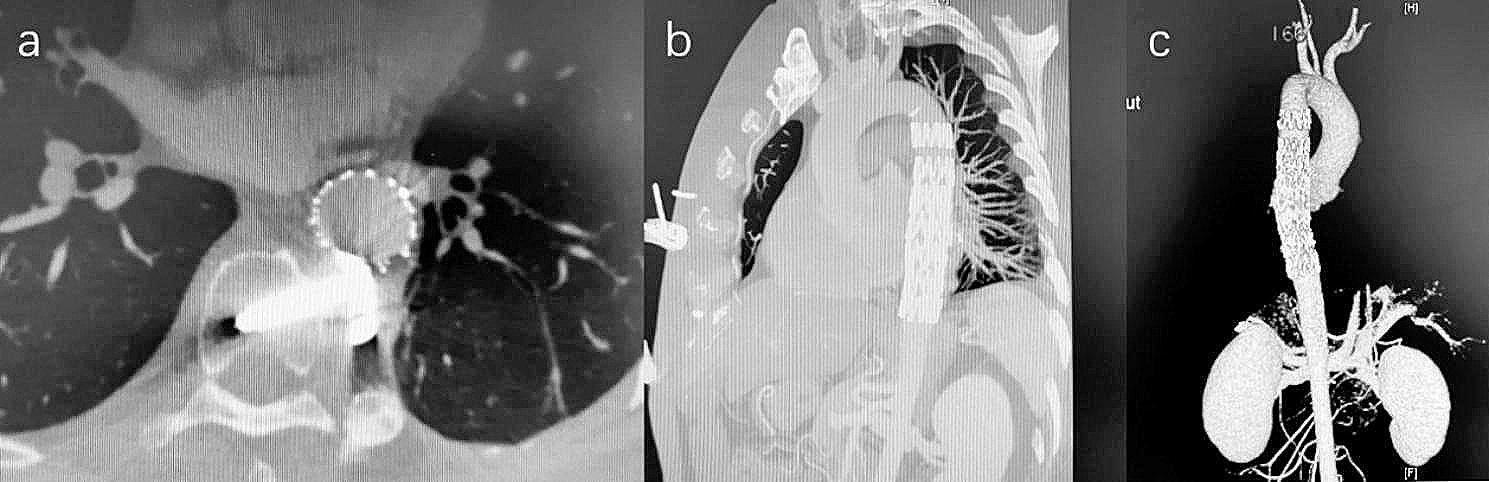
CT angiography revealed complete exclusion of the pseudoaneurysm with no endoleak

Under general anesthesia, the right femoral artery was accessed, and angiography confirmed the pseudoaneurysm in the mid-segment of the descending aorta, just located at the level of the spinal column fixation instruments. No significant AKA was identified during the procedure, and several pairs of intercostal arteries of similar sizes were visible. A 28–80 mm stent graft (Lifetech, Shenzhen, China) was placed via the right common femoral artery to seal the lesion. Subsequent angiography showed slight type Ia endoleak. Considering the risk of endoleak and the potential durability problems caused by friction between the stent graft and the screw, we decided to implant a second stent graft. Subsequently, a 28–140 mm stent graft (COOK, Bloomington, USA) was deployed just inside the first one. The second stent graft lengthened the proximal landing zone and strengthened the local abrasion resistance. The final angiography revealed complete exclusion of the pseudoaneurysm with no endoleak (Fig. 2). Due to the orthopedic consultation determining that the removal would pose significant risks and the patient’s refusal of further open surgery, the screws were not extracted. Extubation was carried out in the operating room under general anesthesia, and assessment of lower limb function was performed. The operation was successful, the patient recovered well and was discharged from hospital 5 days later. There were no complications such as paraplegia, paresis or infection. Postoperative CT scans showed excellent apposition of the two stent grafts with no evidence of endoleak.

During a follow-up period of nearly 8 years, CT scans indicated that the descending aortic stent grafts keep patency, and the lesion became smaller year by year and gradually absorbed. He recovered well and remained asymptomatic. The quality of life has not been affected in any way.

## Discussion

Over time, due to advancements in biomaterials and surgical techniques, there has been a gradual increase in the adoption of segmental pedicle screw implantations for treating spinal deformities, not only in the lumbar spine but also in the thoracic spine. A list of 11 case reports of injuries to thoracic aorta due to the spinal surgeries in the past decade is presented in Table 1. The forms of aortic injuries primarily include penetration and pseudoaneurysm. Among them, 8 patients had aortic penetrating injuries, and 3 patients had pseudoaneurysms. The treatment methods included the placement of covered stents and open surgical procedures. 4 patients underwent open surgery, and 7 patients underwent endovascular repair with placement of covered stents. One patient who had a covered stent placement experienced recurrence of type B dissection after one year, and another patient died from abundant aortic hemorrhage six months after the procedure. The remaining patients recovered well after surgery. Among them, 7 cases were located in the T10-L2 segment. This is because the thoracolumbar spine (T10-L2) is at the intersection of the thoracolumbar physiological curve, which is the area of highest stress concentration and prone to fractures requiring screw fixation. It is worth noting that most of the literature reports had a follow-up time of less than one year, and the longest reported follow-up time in previous literature was five years. The cases reported in this study had a follow-up time of eight years, which is currently the longest known follow-up time.

**Table 1:** Summary of studies on thoracic aorta injuries after spinal surgery

Reference	Type of Spinal Surgery	Surgical Spinal Level	Postoperative Diagnosis Time	Diagnosis	Treatment Approach	Follow-Up Time	Late-Term Outcome
Ip et al., 2014 [1]	T6 vertebrectomy and pedical screw fixation	T5-T7	5 years	Pseudoaneurysm	ESG	6 months	Healing
Pesenti et al., 2014 [2]	Pedical screw fixation	T5-L4	2 days	Penetrating injury	ESG	NK	Healing
Tong et al., 2015 [3]	Pedical screw fixation	T11-L2	17 months	Penetrating injury	ESG	1 month	Healing
Lagios et al., 2015 [4]	Pedical screw fixation	T10-L4	6 years	Penetrating injury	ESG	2 years	Healing
Claiborne et al., 2015 [6]	Pedical screw fixation	T2-S1	15 months	Pseudoaneurysm	ESG	1 year	Recurrence of type B dissection
Sevuk et al., 2016 [7]	Pedical screw fixation	T3-T9	5 years	Penetrating injury	Open repair	NK	Healing
Martin et al., 2018 [8]	Pedical screw fixation	T8-L3	2 weeks	Penetrating injury	ESG	6 months	Healing
Kayacı et al.,2019 [9]	Pedical screw fixation	T10-L1	1 month	Penetrating injury	ESG	6 months	Died from abundant aortic hemorrhage
Kayacı et al.,2019 [9]	Pedical screw fixation	T12-L4	1 day	Penetrating injury	Open repair	1 year	Healing
Kayacı et al.,2019 [9]	Pedical screw fixation	T10-L2	1 day	Penetrating injury	Open repair	8 months	Healing
Liu et al.,2021 [11]	Pedical screw fixation	T11-L2	10 days	Pseudoaneurysm	Open repair	4 months	Healing

ESG, endovascular stent graft; NK, not known

To prevent the occurrence of postoperative vascular complications, it is essential to consider various factors before spinal surgery, such as a history of prior intervertebral disc surgeries and existing vascular disease histories. For instance, patients undergoing vertebral body fixation require an assessment of vascular conditions and the distance between vessels and vertebrae to determine the extent to which vessels can safely reach the required distraction for device placement. Additionally, atherosclerosis increases the risk of vascular complications due to the loss of arterial elasticity and recoil [10]. At the same time, meticulous intraoperative procedures and, if necessary, intraoperative ultrasound guidance are performed to avoid vascular injury during surgery.

Early complications are often identified by hypotension, tachycardia, active bleeding, a taut abdomen, the presence of an abdominal mass and/or abdominal pain. The most common late complications are arteriovenous fistula(AVF) and pseudoaneurysm. It has been reported that most of these cases are recognized within 18 months after spinal surgery [12]. Henk et al [13] reported a case where a patient developed a pseudoaneurysm of the thoracic aorta 20 years after undergoing anterior spinal fixation surgery. For patients who exhibit signs of hypovolemic shock during surgery, vascular imaging can be used to confirm the type and location of the vascular injury. The diagnosis of late complications can be challenging, the pseudoaneurysm can lead to compressive symptoms related to adjacent structures, such as the trachea, esophagus, superior vena cava, or phrenic nerve. Secondary fistulas may develop and result in different symptoms like hemoptysis, hematemesis, or life-threatening hemorrhage. If a patient presents with these symptoms, vigilance should be maintained for the emergence of pseudoaneurysms, arteriovenous fistulas, and other late complications. Vijay et al [14] reported a case in which a vascular anomaly was misdiagnosed as an infection, emphasizing the need for surgeons to be aware that most bleeding pathways from pseudoaneurysms can lead to secondary infections. Additionally, early postoperative diagnosis using techniques such as Doppler ultrasound, digital subtraction angiography, CT scans, and MRI (magnetic resonance imaging) scans is crucial.

Treatment options can generally be divided into open surgery, endovascular repair, and hybrid surgery (combining open surgery with stent placement). Of course, treatment options are not limited to these three methods. Geert Maleux et al [15] reported a case of recurrent pseudoaneurysm of the aorta following failed open surgical repair that was treated with thrombin injection via the lumbar spine. In the 21st century, there has been an increasing use of endovascular repair with good mid- to long-term follow-up results. Endovascular techniques have continuously advanced and can now address most issues with fewer complications compared to open surgery. However, Bavare et al. [16] reported a patient presented with hemoptysis due to aortic erosion between the spinal internal fixation device and an early-placed endovascular stent graft, necessitating a surgical intervention. Due to the friction between the covering stent and the internal fixation device, we can consider utilizing a more secure covering stent, even a double-layered covering stent, to maintain long-term repair effects. Annual long-term follow-up CT scans are optimal for monitoring, and in the event of any signs of local stent damage, re-stenting or open surgery can be considered. Re-stenting is less complex and repeatable. This is the significance of our 8-year long-term follow-up.

## Conclusion

Vascular injury caused by spinal instrumentation can have fatal consequences and should be promptly diagnosed and managed. Prevention should also be emphasized, and spinal surgeons should have a thorough understanding of the local anatomy and carefully study the relative positional relationship between the vertebral bodies and the major vessels at the surgical site. For high-risk cases of vascular injury, intraoperative ultrasound assessment may be considered to allow for timely adjustment of the position of the internal fixation device. In cases where vascular injury signs occur intraoperatively or during follow-up, diagnostic imaging should be used. Once the diagnosis is confirmed, open surgery and stent placement are both viable treatment options, and individualized surgical decision-making remains paramount. With advances in endovascular techniques, if the lesion does not involve important branches of the aorta, covered stent endovascular repair can lead to faster recovery, and the current evidence suggests satisfactory mid- to long-term follow-up outcomes.

## Data Availability

No datasets were generated or analysed during the current study.

## Abbreviations

CT: Computer tomography
MRI: Magnetic resonance imaging
